# Advance Care Planning and Hospice Use Among People with Dementia: A Report from the Health and Retirement Survey

**DOI:** 10.1093/geroni/igab046.3352

**Published:** 2021-12-17

**Authors:** Kathryn Coccia

**Affiliations:** Saint Louis University, St. Louis, Missouri, United States

## Abstract

People with Alzheimer’s disease and related dementias (ADRD) frequently receive sub-optimal end-of-life care (EOLC), often enduring invasive procedures such as tube feeding, resuscitation, and surgery within days of their death. While advance care planning (ACP) has shown effectiveness in improving EOLC for those with ADRD, there are many barriers to ACP specific to the ADRD population. Research suggests that hospice care is optimal in reducing end of life suffering for ADRD patients. This study aimed to empirically assess hospice utilization and ACP for individuals with ADRD compared to individuals without ADRD, and to assess the impact of ACP on hospice utilization for individuals with ADRD. Data came from the 2016-2018 wave of the Health and Retirement Study (HRS), a national longitudinal study collecting health and demographic data on older Americans. This analysis evaluated survey responses from 1,224 proxy respondents for individuals who died during this period. In this sample, people with ADRD were both significantly more likely to have utilized hospice care (OR=1.37) and to have written EOLC instructions in place (OR=1.19). Those with ADRD were 22% less likely to have discussed their EOLC wishes with their proxy than those without ADRD. Having a written EOLC plan in place significantly increased the odds of hospice utilization (OR=1.37) but discussion around EOLC preferences increased odds of hospice utilization at a higher rate (OR=1.59). These results support policy to advance earlier ACP conversations around EOLC preferences and the implementation of written EOLC instructions to reduce suffering for individuals with ADRD diagnoses .

